# Using a Group Stimulus Preference Assessment to Design an Effective Group Contingency

**DOI:** 10.1007/s40617-024-01003-2

**Published:** 2024-10-17

**Authors:** Amber S. E. Fluharty, Lauren M. LeJeune, Mark D. Samudre

**Affiliations:** 1 Anderson School District One, Anderson, SC USA; 2 University of South Carolina, 820 Main St., Columbia, SC 29208 USA; 3 University of Kentucky, Lexington, KY USA

**Keywords:** Group contingency, Middle school, Preference assessment, Single case design, Special education

## Abstract

The purpose of this study was to extend the limited research on group stimulus preference assessment (SPA) procedures. The study occurred in two sections of a middle school special education classroom and included 20 students with multi-categorical disabilities. A paired stimulus group SPA was used to identify a preference hierarchy for each class, and results were validated using single case designs in which baseline conditions were compared to group contingency conditions with alternating sessions of the highest-preferred (high-p) and lowest-preferred (low-p) consequences. Both group contingency conditions increased the percentage of students prepared for class relative to baseline; however, consistently higher levels of students were prepared for class during high-p sessions. Student responses on a daily quiz were similar in both conditions, but social validity surveys indicated students felt the most motivation and enjoyment during high-p sessions.

**Supplementary Information:**

The online version contains supplementary material available at 10.1007/s40617-024-01003-2.

A group contingency is a proactive and efficient classroom management strategy that teachers can embed within naturally occuring classroom activities to increase desired behaviors of all students in the room (Chow & Gilmour, 2015; Ennis, 2018; Maggin et al., 2012). The broad components of a group contingency include establishing shared classroom expectations, explicitly teaching those expectations, identifying students who meet the expectations (e.g., awarding points), and then reinforcing those students’ behavior with a shared consequence (e.g., providing a break or tangible reward; Chow & Gilmour, 2015; Pokorski et al., 2017). Group contingencies are categorized as: (a) *independent,* if each student who meets the expectations receives the reward; (b) *interdependent;* if all students in the group must meet the expectations for everyone to receive the reward; or (c) *dependent,* if a certain students must meet the expectations for all students to receive the reward (Pokorski et al. 2017). Although researchers have evaluated packaged group contingencies that teachers may replicate [e.g., Good Behavior Game (Barrish et al., 1969) or Classwide Function-Related Intervention Teams (Wills et al., 2009)], teachers may also design unique group contingencies to meet the specific needs of their students and classroom context (Chow & Gilmour, 2015; Maggin et al., 2012; Pokorski et al., 2017).

Multiple systematic reviews have supported the general effectiveness of group contingencies in various settings. For example, Maggin et al. (2012, 2017) conducted two reviews of group contingency research in which they identified 121 total single case design studies that occurred in K–12 classrooms and included students with high-incidence disabilities (e.g., learning disabilities, behavioral disorders). Although Maggin et al. concluded group contingencies are an evidence-based practice, the studies identified within these reviews primarily occurred in general education settings and excluded participants with autism or intellectual disability. Additional reviews have provided support for the use of group contingencies in other contexts, including preschool classrooms (Pokorski et al., 2017) and alternative schools for student with behavioral disorders (Groves et al., 2023). Nonetheless, there is a need for research on the use of group contingencies in special education classrooms that include students with a broader range of disabilities.

Systematic reviews have also highlighted a need for additional research on the effects of independent group contingencies. For example, Maggin et al. (2012, 2017) exclusively identified studies that used interdependent or dependent contingencies, and Pokorski et al. (2017) found that only three of ten studies in their review included independent contingencies. Independent group contingencies may be preferable in certain situations, such as when teachers are concerned that one or more students could sabotage the group if an interdependent group contingency were implemented (Groves et al., 2023). A small number of recent studies indicate that independent contingencies can decrease disruptive behaviors and increase appropriate behaviors (e.g,. Joslyn et al., 2023; Pasqua et al. 2021) and may have similar effects as interdependent contingencies (Groves & Austin, 2017). These promising findings indicate that further studies of independent group contingencies are warranted.

## Methods for Predicting Reinforcers

A key component of group contingencies is providing a valuable reinforcer to the students who meet expectations (Chow & Gilmour, 2015; Ennis, 2018; Maggin et al. 2012). Given the idiosyncratic nature of reinforcement, identifying a reward that is likely to motivate an entire group of students can be challenging (Radley et al., 2019). Nonetheless, Ennis (2018) recommended that teachers can select reinforcers by: (a) polling students to select a group reward, (b) allowing each student to select their own reward, or (c) using a teacher-selected reinforcer that is either shared or kept secret from the class. Teachers might also use stimulus preference assessments (SPAs; Cooper et al., 2020) to identify potential reinforcers; however, SPAs are typically conducted in one-on-one formats. Group contingency research has rarely reported the use of direct SPAs (Pokorski et al., 2017), potentially due to the time required to conduct an SPA with each student and synthesize results for the group (Radley et al., 2019). Although group contingencies are considered generally effective without the use of direct SPAs (Maggin et al., 2012; Pokorski et al., 2017), the inclusion of preferred reinforcers may further increase their efficacy and result in socially valid intervention procedures (Lill et al., 2021). Adapting SPA procedures to a group format may result in an efficient method for predicting reinforcers to include within group contingency interventions.

Conducting an SPA involves three general steps: (1) identifying a pool of stimuli (e.g., small toys or activities) that could be feasibly used as reinforcers in the context; (2) systematically presenting the options to the learner (i.e., in a trial-based format) or allowing the learner to interact freely with all options (i.e., in a free operant format); and (3) analyzing results to establish a hierarchy of highest-preferred to least-preferred options (i.e., a preference hierarchy; Livingston & Graff, 2018). It is important to note that stimuli selected as highly preferred during an SPA may not actually increase desirable behaviors when presented contingently and should be considered *potential reinforcers* (Lill et al., 2021). The reinforcing value of preferred stimuli can be confirmed through systematically comparing conditions in which the stimulus is either (a) presented or (b) withheld contingent on a target behavior (i.e., reinforcer assessment; Karsten et al., 2011). However, research indicates that preferred stimuli function as reinforcers often enough that teachers can justifiably omit a more effortful reinforcer assessment (Cooper et al., 2020; Lill et al., 2021).

Researchers have developed variations of the general SPA procedures to fit the characteristics of learners and their environments, resulting in multiple SPA options (e.g., free operant, trial-based) from which practitioners can choose (Cooper et al., 2020; Lill et al., 2021). Two of the trial-based options—paired stimulus (PS; Fisher et al. 1992) and multiple stimulus without replacement (MSWO; DeLeon & Iwata, 1996)—are most likely to result in identification of a preference hierarchy (Karsten et al., 2011; Lill et al., 2021). These assessments have traditionally involved placing tangible objects in front of the learner (2 items for PS and 5–7 items for MSWO), allowing the individual to interact with the chosen object, and then repeating trials until all choices are presented or the learner stops selecting items (Karsten et al., 2011; Cooper et al., 2020). Research has indicated high agreement between the results of MSWO and PS formats (DeLeon & Iwata, 1996; Lill et al., 2021).

Researchers have also identified efficient SPA variations in which individuals were not given immediate access to the chosen stimulus (e.g., Brodhead et al., 2019; Daly et al., 2009) and paper or electronic pictures replaced physical items (e.g., Brodhead et al., 2016; Lill et al., 2021; Radley et al., 2019). Pictorial versions of these assessments are likely appropriate when students can discriminate between two or more stimuli and can match objects to pictures (Lill et al., 2021). The MSWO has been recommended as a first choice for students with these characteristics due to its relative efficiency (Karsten et al., 2011); however, teachers may prioritize other contextual factors when choosing an SPA (e.g., student, setting, or stimulus characteristics; Lill et al., 2021).

## Previous Research on Group SPA Procedures

There is currently limited research on group SPAs. In one example of an indirect group SPA, Resetar Volz and Cook (2009) administered a preference survey to 313 children and adolescents within a residential facility. The survey included options from multiple preference categories (e.g., edible, activity) and participants rated their preference for each option using a Likert-type scale. The indirect survey method allowed researchers to establish a hierarchy of client-reported preferences; however, it is unknown whether results would correspond with a direct SPA or result in an effective behavioral intervention.

In one example of a direct group SPA, Layer et al. (2008) developed a multiple-stimulus procedure with 14 preschoolers without disabilities. The researchers first assessed participant preference for small edible items in an individual format with immediate access to the item available after selection. Then procedures were adapted to a group format in which: (1) participants were arranged in groups of three, (2) each group member selected an edible option from a picture, (3) the group member’s choice was placed in a voting box, and (4) access to one of the edibles was available based on random selection from the box. Results from the two SPA formats were similar for each child (i.e., participant preference was either variable or consistent regardless of format) and the group format resulted in more efficient identification of preference.

In an extension of the Layer et al. (2008) study, Radley et al. (2019) evaluated individual and group versions of a multiple stimulus with replacement SPA with 19 students in a 7th grade general education classroom. Individual sessions were similar to those conducted by Layer et al. During the group SPA, students chose from pictorial options using online survey software. Following each trial, the software generated a spinner wheel with sectors sized in accordance with student choices. The wheel was spun and students received access to the stimulus identified. Results were similar to previous research in that the group SPA resulted in clearer identification of preferences compared to the individual method. However, neither Layer et al. nor Radley et al. included a reinforcer assessment or intervention evaluation. Thus, it is unknown whether their group SPA procedures accurately predicted reinforcers for increasing desirable behaviors.

In sum, a small group of studies indicate that results of group SPAs may correspond with individual SPA results, and group SPAs can result in efficient identification of preference hierarchies for groups of 3 to 19 students in general education settings. However, this research was limited to multiple stimulus formats that included immediate access to the chosen stimulus. There is a need to evaluate alternative group SPA formats that may better align with specific student, settings, and stimulus characteristics. For example, a PS format may be appealing to teachers because, unlike an MSWO format, it would not require them to count votes during the assessment to determine which stimuli to remove between each trial. Previous research was also limited to students without disabilities and did not evaluate whether group SPAs can accurately predict reinforcers for inclusion in a behavioral intervention. Development of direct SPA procedures to predict reinforcers for group contingency interventions would have important implications for special educators who support the behavior of students with disabilities in group settings.

## Purpose and Research Questions

The purpose of this study was to extend the limited research supporting the use of group SPA procedures by including students with disabilities, developing a PS format of the group SPA, and validating results in the context of a behavioral intervention. Assessments and intervention occurred during social studies instruction in a multi-categorical middle school special education classroom with the ultimate goal of increasing the number of students prepared for class. Our primary research questions were: (1) will a group SPA result in identification of a preference hierarchy for middle school students with disabilities, as measured by the percentage of votes allocated to each choice? and (2) During group contingency sessions, is there a consistent difference in the percentage of students prepared for class when the highest-preferred (high-p) consequence is available compared to when the lowest-preferred (low-p) consequence is available? In addition to our experimental questions, we also tracked student responses on a daily quiz and surveyed students about their preference for intervention conditions (i.e., high-p versus low-p sessions).

## Method

### Participant Recruitment

The participants in this study were middle school students with disabilities. Participants met the following criteria: (a) identified with an educational disability by a school district [e.g., autism spectrum disorder (ASD), intellectual disability (ID), specific learning disability (SLD), multiple disabilities]; (b) received at least 1 hr of special education services daily outside of the general education setting; and (c) had a parent’s signed consent to participate. We received approval from a university-based institutional review board and a school district in the Southeastern United States, as well as signed consent from the middle school principal, before recruiting participants. We then sent consents home to the parents of 26 students from two sections of a special education classroom. Two parents declined for their child to participate, and four parents did not return the consent form. These students participated in study activities with the rest of their class, but their data are omitted.

### Participant Characteristics

There were 20 total student participants who ranged in age from 11 to 14 years old. All students communicated by speaking in full sentences, could follow spoken directions, and could read text at a Kindergarten or higher level. Classroom One included 12 students, of which four were girls and eight were boys. Five of the students were White, two students were Black, and five students were Hispanic. Primary disability categories included SLD (*n* = 7), ASD (*n* = 4), and other health impairment (OHI; *n* = 1). Four of the 12 students were classified as an English-Language Learner (ELL).

Classroom Two included eight students, of which three were girls and five were boys. Two of the students were White, five students were Black, and one student was Hispanic. Primary disability categories included SLD (*n* = 5), OHI (*n* = 2), and ID (*n* = 1). One student in Classroom Two was also classified as an ELL.

### Setting and Study Timeline

The setting was a self-contained middle school special education classroom for students with multi-categorical disabilities. The classroom was designed for students with low support needs who were on track to receive an employability credential upon completing high school (i.e., non-diploma track), and the teacher provided modified grade-level instruction. All SPA, baseline, and intervention sessions occurred during social studies periods. Individuals present in the room included students, the teacher, and the instructional assistant. Students were seated at individual desks arranged in rows facing toward an interactive whiteboard. We completed all study activities with Classroom One across two months (i.e., Oct–Nov) and then replicated procedures with Classroom Two during the subsequent two months (i.e., Dec–Jan).

### Implementer and Research Team

This study occurred within the context of a graduate-level university practicum designed to meet the Behavior Analyst Certification Board’s (BACB) requirements for concentrated supervised fieldwork. The implementer was a graduate student who was completing her fourth semester of courses and was concurrently working as a full-time special education teacher. This study occurred in her classroom. She was a 32-year-old White, Hispanic female who held a Master of Arts in Teaching Elementary and Special Education Learning Disabilities and had been teaching for 6 years. She had completed didactic coursework in SPA procedures and single case research design, and she piloted the group SPA during a previous semester of practicum. She is referred to as “the teacher” in all subsequent sections.

One instructional assistant in the classroom served as a secondary data collector. He was a 30-year-old, Black, Non-Hispanic male who had an associate’s degree in human services and had been in his position for 5 years. Training procedures and interobserver agreement (IOA) results are described in the Dependent Variable section.

The design and implementation of study procedures was supervised by a university practicum instructor. She was 31-year-old White, non-Hispanic female who had a Ph.D. in Special Education and was a Board Certified Behavior Analyst (BCBA). She provided individual supervision for 1 hr/week throughout the study, which included: (a) input on all study procedures and data collection forms, (b) rehearsal of procedures with feedback, (c) weekly review of participant data, and (d) performance feedback following fidelity checks. The supervisor also guided the teacher through all experimental decisions (i.e., when to change phases).

### Group SPA Procedures

We developed a group SPA using a PS format and electronic pictures to represent available options. The teacher implemented three sessions of the group SPA over three days (i.e., one per day) and sessions were approximately 15 min long. Materials included: (a) charged Chromebooks for student use, (b) a Google Slides deck with paired pictures of options, (c) an interactive whiteboard to display the slides at the front of the classroom, and (d) a Google Form for collecting data on student votes. Participants met the characteristics recommended for both pictorial versions of MSWO or PS formats (Lill et al., 2021); however, the teacher chose a PS format because she hypothesized it would be more feasible for her classroom. Specifically, an MSWO format would have required the teacher to count student votes immediately and then remove the most preferred option; whereas the PS format allowed her to present all options without interruption (using pre-made slides) and then count total votes after each session.

We selected a range of stimuli that were feasible to use as reinforcers in the classroom setting and had been commonly available during leisure times since the beginning of the school year. Specific considerations included: (a) the ability to deliver the reinforcer to students who met the contingency while withholding it from those who did not, (b) activities that required no more than 10 min during the 50-min class period, and (c) no edibles were allowed due to student diet restrictions. On the basis of these considerations, the teacher identified seven total stimuli: (1) an online computer game (i.e., Blooket); (2) free time on Chromebooks; (3) drawing; (4) walking a lap on the school’s running track (i.e., walking the track); (5) a social game (i.e., Heads-Up 7-Up); (6) sorting classroom items by color; and (7) a break without access to tangibles (i.e., head down on desk). We also included the option for students to choose neither activity on each slide to align with traditional SPA procedures in which a learner may reject all of the available options (Fisher et al., 1992; DeLeon & Iwata, 1996).

Group SPA sessions followed five steps. First, the teacher posted the Google Form to Google Classroom. Second, the teacher introduced a game called “This or That Reward Edition” and displayed the Google Form on the whiteboard. The form was formatted as a one-page survey that included a box for students to type their initials and boxes of multiple-choice questions aligned with each slide of the presentation. Immediately prior to the first session only, the teacher displayed the slides and survey questions side-by-side on the whiteboard and verbally described how to choose an option from a slide and then select the corresponding option on the survey. Third, the teacher asked students not to say their choices aloud or to look at other students’ computers. Fourth, the teacher displayed each slide while reading the choices aloud and students used the Google Form to select a choice. For example, the second slide displayed a picture of the Blooket game with “Choice A” written underneath and a picture of a Chromebook with “Choice B” written underneath. The first question on the Google Form said, “Slide 2 This or That” and included radio buttons for Choice A, Choice B, or Neither. Students used the track pad on their Chromebooks to select one of the available options. We counterbalanced the position of choices (i.e., from right to left) across slides and used the same slide deck for all three sessions. The students used a thumbs up to indicate when they were done selecting a choice and were ready for the teacher to move to the next slide. This step was repeated for 21 total choices. Fifth, the students submitted their completed Google Forms through Google Classroom.

The supervisor collected fidelity data during one of the three group SPA sessions (33%) for each class by directly observing through Zoom video conferencing. Data were collected using a yes/no checklist of assessment steps that occurred once (e.g., introducing the game, asking students not to share their choice) and a per opportunity method for implementing each of the 21 trials (i.e., stating the two choices and waiting for students to indicate completion). Results indicated 100% fidelity for both classes.

## Dependent Variables and Data Collection

### Group SPA

To answer research question one, we collected data on the percentage of total votes each choice received during three group SPA sessions. After each class completed three sessions of the SPA, we downloaded their responses to a spreadsheet through Google Classroom. We calculated the total votes each item received, divided that value by the total number of votes cast, and multiplied by 100 to derive a percentage. We defined the high-p choice as the one which received the highest percentage of votes across three sessions; conversely, the low-p choice received the lowest percentage of votes. We also calculated session-by-session data to allow for an analysis of data stability across administrations.

### Group Contingency

#### Being Prepared for Class

To answer research question two, we collected data on the percentage of students who were prepared for class during group contingency sessions. This was an area of need within the classroom because many of the students did not have necessary materials which often led to low student engagement and an increase in off-task and disruptive behavior throughout the class period. We defined *being prepared* as students sitting at their desk, without talking, facing the front of the classroom, with materials on their desk when the bell rang at the start of class (i.e., 9:00 AM and 10:00 AM). The two required materials (i.e., a pencil bag and spiral notebook) were always available in a classroom bin located on a table opposite of the door where students entered. Examples of being prepared included students sitting with their bottom on the chair and feet on the floor and students having their pencil bag and spiral notebook on top of their desk. Non-examples included students missing their pencil bag or notebook from the top of their desk, walking around the classroom, standing near their desk, turned toward the back of the room or toward the window, still gathering materials after the bell rang, sitting on the desk or with their legs under their bottom, or talking.

The teacher used a checklist to collect data on the number of students prepared at the start of each session (i.e., immediately after the bell rang). Students’ initials were listed on the data sheet in the order of their desk in the classroom. The teacher stood in the front of the classroom and started to collect data at the left of the room. For each student, she recorded a checkmark if they were prepared or an X mark if they were not prepared. At the end of the session, the teacher calculated the percentage of students who were prepared by dividing the number of students prepared by the total students present that day and multiplying by 100. Thus, these data represent the percentage of students who were prepared given one opportunity.

#### Interobserver Agreement

In addition to the teacher, one instructional assistant collected data on the percentage of students prepared. The teacher trained the assistant during one behavioral skills training (BST; Parsons et al., 2012) session. First, she described the operational definition of being prepared and modeled examples versus non-examples. Second, she described the data collection steps, modeled data collection, and gave him the opportunity to practice while providing descriptive praise and corrective feedback. Prior to independent data collection, the two data collectors practiced taking data in the classroom until achieving 100% agreement for three sessions.

We aimed to measure interobserver agreement (IOA) for at least 20% of sessions in each phase and condition and achieved this for Classroom One. However, we omitted the baseline phase from IOA data collection for Classroom Two in error. We calculated IOA using a point-by-point method in which we compared the teacher and assistant’s data collection for each student. We calculated the percentage of agreement by dividing the total number of agreements by the total number of students and multiplying by 100. Mean IOA was 97.29% (range = 81–100%) for Classroom One (25% of baseline sessions; 38.46% of intervention sessions) and 100% for Classroom Two (25% of intervention sessions).

#### Correct Responses to Daily Quiz

As a secondary variable, we also collected data on students’ responses to a daily quiz. The purpose of including this variable was to provide descriptive information about whether being prepared was associated with academic responding (i.e., social validation). Accordingly, quiz responses were not linked to the group contingency or experimental decisions. The teacher created a fill-in-the blank question with four choices (i.e., the correct answer and three plausible distractors) to assess recall of facts from the news video students watched at the start of class. For example, one question stated, “According to CNN10, in Indonesia, a deadly _______ erupted and covered some Javanese villages in a thick coat of ash,” with choices: (a) volcano, (b) tornado, (c) mudslide, and (d) hurricane. The teacher gave students a slip of paper with the quiz after they watched a 2- to 4-min news segment. The students responded by circling the letter next to one of the options, and the teacher scored the quizzes at the end of each class period using a pre-developed answer key. We then calculated the percentage of correct responses per session by dividing the total number of correct responses by the number of students present and multiplying by 100.

### Experimental Design

With Classroom One, we used an ABAB design with an embedded alternating treatments design (ATD) to evaluate the effect of the group contingency on student behavior and to compare student behavior during high-p versus low-p conditions. We based experimental decisions on the percentage of students prepared for class. Baseline observations occurred for at least three sessions or until data were stable. During intervention phases, the reinforcer for the group contingency alternated between high-p sessions and low-p sessions based on a block randomization schedule. Specifically, we randomly chose the option available in the first session of each block and then assigned the other option to the second session in the block. We implemented at least three sessions per condition (i.e., six per phase).

We initially planned to use the same experimental design with Classroom Two; however, we had insufficient time to complete all phases within the study timeline. Thus, we included one baseline phase and one ATD phase. This allowed us to experimentally compare the effect of high-p versus low-p conditions in Classroom Two; however, we were unable to demonstrate the overall effect of the group contingency on the behavior of students in Classroom Two.

## Group Contingency Procedures

### Baseline

Baseline sessions began after the group SPA was complete. At the start of each baseline session, the teacher set the student notebook and pencil bins on a table in the front of the classroom. The expectation to enter quietly, gather supplies, and take a seat before the bell rang (i.e., being prepared) had been described at the beginning of the school year. However, no prompts were provided during baseline sessions and students did not know data collection was occurring. After the bell rang, the teacher began normal instructional procedures which consisted of a current event news video followed by social studies instruction. The instructional assistant stayed within close proximity of students and provided occasional behavior corrections. During the second baseline phase (for Classroom One only), the teacher reinstated these same procedures without explicitly stating that reinforcement was unavailable.

After the first segment of the news video (i.e., 2–4 mins), the teacher paused the video and administered the daily quiz. The teacher read the quiz question multiple times while circulating the room. The students were told to put the date on the slip and their initials, and then to select the best answer and flip over the slip when done. After all quizzes were complete and collected, the teacher went over the answer with the class and resumed the news video.

### Group Contingency

We designed an independent group contingency in which each student could access the scheduled consequence based on whether they were prepared for class (Pokorski et al., 2017). High-p versus low-p consequences were scheduled using the procedure described in the Experimental Design section. Research indicates group contingencies may be most effective when classroom rules are actively taught as part of the intervention package (Maggin et al., 2012). Thus, the day before starting the group contingency, the teacher shared a task analysis for being prepared and re-taught students the steps by modeling and giving them the opportunity to practice and ask questions. Winter break occurred after the initial teaching session for Classroom Two; therefore, the teacher conducted another teaching session with the same procedures before beginning intervention.

For each intervention session, the following contingency was posted on the whiteboard: “In order to earn [the high-p or low-p consequence] you must be prepared for class; otherwise, you will begin instruction.” The teacher read the contingency three to five times as the students entered the room. Upon the bell ringing, the teacher scanned each row and collected data on students who were prepared. The teacher then followed procedures for the news video and the daily quiz as in baseline.

After the 10-min news video was over, the teacher displayed a table with the group contingency results on the whiteboard. All students who were prepared at the beginning of class were listed in a reward column and students who were not prepared were listed in a classwork column. Students who met the contingency then immediately accessed the scheduled consequence for up to 10 min (for Classroom One, one round of the game chosen as the high-p option took 7 min to complete). To ensure that students who did not meet expectations did not also access a break (i.e., differential reinforcement), those students were instructed to independently complete an instructional activity. The teacher and instructional assistant circulated the room to ensure that the students were on task with their assigned activity, and classroom instruction resumed after the reward activity.

### Social Validity Survey

At the conclusion of the study, the teacher administered a student survey with five questions (Table 1) related to their motivation for being prepared for the high-p and low-p conditions. The students had individual access to a Google Form with the survey questions, and during survey administration, the teacher displayed the form on the whiteboard. Prior to starting, the teacher asked students not to look at the Chromebook screens of their classmates and to give a thumbs up when they completed each question. Then she read each question and the answer choice aloud. Once all thumbs were displayed, the teacher read the next question. This procedure continued across all five questions.

**Table 1:** Social validity survey data

Question	Never	Sometimes	Always	Yes	No
1. How motivated were you to be prepared for class before being offered a reward?	1	10	5	–	–
2. How motivated were you to be prepared for class when you could a [high-p reward]?	1	4	11	–	–
3. How motivated were you to be prepared to class when you could earn [low-p reward]?	2	8	6	–	–
4. Do you like working for [high-p reward]?	–	2	–	13	1
5. Do you like working for [low-p reward]?	–	8	–	6	2

*Note.* Results represent the total number of students in both classes who responded to the survey (*n* = 16). Dash marks indicate options that were not available for the given question

## Results

### Classroom One

**Fig. 1:**
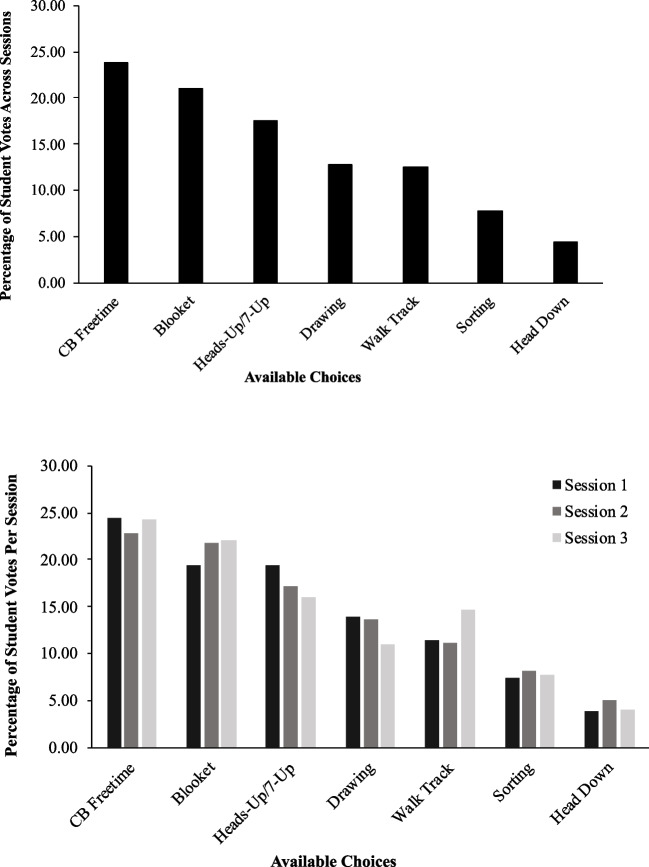
Classroom one group SPA data* . Note.* Data represent the percentage of student votes each choice received out of the total number of votes cast. The top panel depicts a summary of three group SPA sessions and the bottom panel depicts session-by-session data. *CB* = Chromebook

Group SPA data for Classroom One are displayed in Fig. 1, with the top panel depicting summary data from all three SPA sessions. Free time on students’ Chromebooks received 23.86% of votes and was used as the high-p consequence during group contingency sessions. Students allocated the lowest percentage of votes to putting their heads down (4.38%); thus, it was used during low-p sessions. Blooket received a relatively high percentage of votes (21.10%); playing Heads-Up 7-Up, walking the track, and drawing received between 12.50 to 17.53% of votes; and sorting was relatively low-preferred (7.79% of votes). The bottom panel of Fig. 1 depicts session-by-session results. Preference hierarchies were similar across sessions. For all three sessions, free time on Chromebooks was the high-p choice (range = 22.84–24.38% of votes) and head down was the low-p choice (range = 3.98–5.08% of votes).

**Fig. 2:**
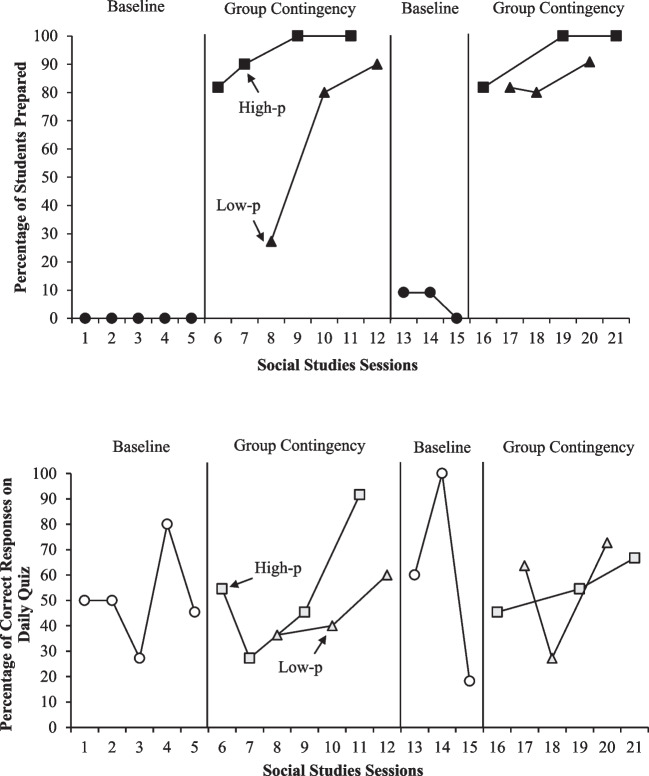
Classroom one group contingency data

Group contingency data for Classroom One are displayed in Fig. 2. During the first baseline phase, no students (0%) were prepared for class during any of the sessions. After implementing the group contingency, the data increased in level and trend for both the high-p and low-p conditions. There was a consistently higher percentage of students prepared in the high-p condition (high-p mean = 92.95%; low-p mean = 65.76%). All students (100%) were prepared during the last two high-p sessions of the first intervention phase. When we returned to baseline for three sessions, the data immediately decreased in level and displayed a decreasing trend with 9.09% to 0% of students prepared. During the second intervention phase, the immediate increase in level across both conditions was replicated. The high-p condition was again associated with more students being prepared, and all students were prepared in the final two high-p sessions. In sum, more students were prepared for class in both group contingency conditions relative to baseline. The high-p condition was also associated with more students prepared than the low-p condition. However, the increasing trend present in low-p sessions during both intervention phases should be noted.

The bottom panel of Fig. 2 depicts data from Classroom One’s daily quizzes. During the initial baseline, quiz responses were variable and ranged from 27.27 to 80% correct. During the first group contingency phase, more correct quiz responses were observed in the high-p condition compared to the low-p condition (high-p mean = 54.74%; low-p mean = 45.45%), though both data paths demonstrated an increasing trend. The data were again variable in the second baseline phase (range = 18.18–100%). In the second intervention phase, data in the high-p condition were variable and decreasing by the end of the phase (mean = 55.56%; range = 45.45–66.67%), and data in the low-p condition were also variable (mean = 54.55%; range = 27.27–72.73%). Overall, visual analysis does not suggest a clear relation between either condition of the group contingency and students’ quiz scores in Classroom One.

### Classroom Two

**Fig. 3:**
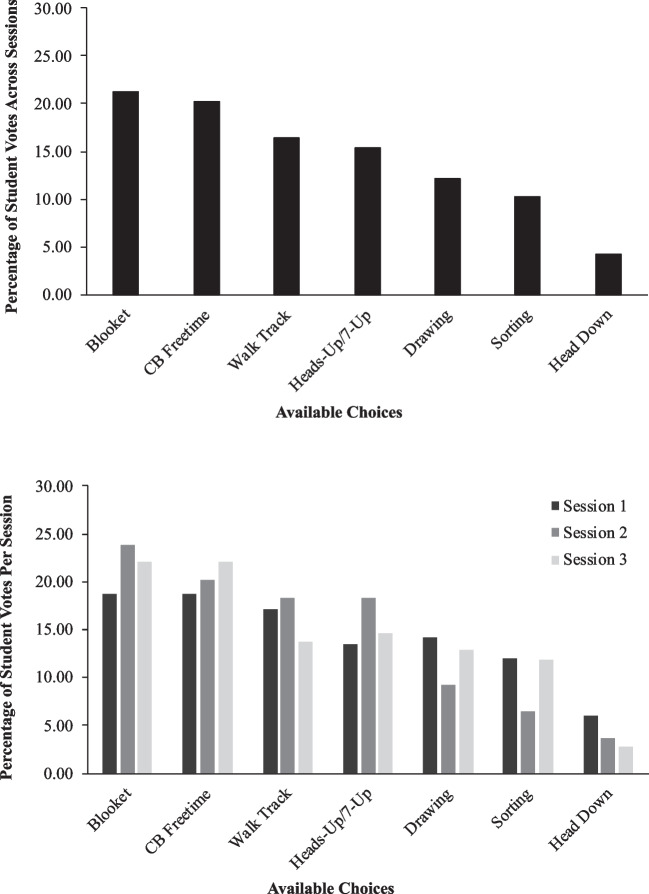
Classroom two group SPA data. *Note*. Data represent the percentage of student votes each choice received out of the total number of votes cast. The top panel depicts a summary of three group SPA sessions, whereas the bottom panel depicts session-by-session data. *CB* chromebook

Figure 3 depicts group SPA data for Classroom Two. Across all three sessions combined (top panel), the high-p choice was playing Blooket (21.31% of votes) and the low-p choice was heads down (4.26% of votes). Chromebook free time received the second highest percentage of votes (20.17%) and the remaining choices received between 10.23 and 16.48% of votes. For each individual session (bottom panel), preference hierarchies were similar. However, Blooket and Chromebook free time received equivalently high percentages of votes during Sessions 1 and 3 (18.66% and 22.02%; respectively) and thus were “tied” as the high-p choice during those sessions. Heads down was the low-p choice for all sessions (range of votes = 2.75–5.97%).

**Fig. 4:**
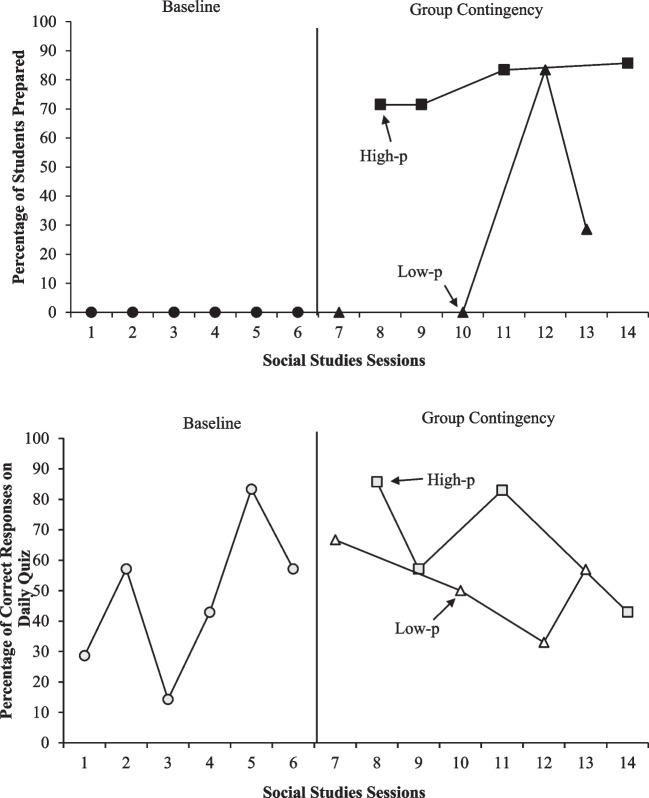
Classroom two group contingency data

Figure 4 depicts group contingency data for Classroom Two. During baseline, no students were prepared for class during any of the sessions. After implementing the group contingency, the percentage of students prepared for class immediately increased for the high-p sessions (mean = 77.98; range = 71.43 to 85.71%) and was variable for the low-p sessions (mean = 27.98; range = 0 to 83.33%). Overall, consistently more students were prepared for class during group contingency sessions in which high-p consequences were available. However, the variability of data in the low-p condition suggest an increasing trend might have emerged with additional data collection.

The bottom panel of Fig. 4 depicts data from Classroom Two’s daily quizzes. During baseline, correct responses were variable with an overall increasing trend (range = 14.29 to 83.33%). During the group contingency phase, more correct responses were observed overall in the high-p condition (high-p mean = 67.21%; low-p mean = 51.67%), but there was considerable overlap between the two conditions. Thus, a relation between either condition of the group contingency and students’ quiz scores was not apparent in Classroom Two.

### Social Validity Survey

Table 1 displays the results of the social validity survey with responses for both classrooms combined. Most respondents (62.5%) stated they were *sometimes* motivated to be prepared for class prior to being offered a reward. Most participants stated they were *always* motivated (68.75%) on the high-p session days and *sometimes* motivated (50%) on the low-p session days. When asked if they liked working for rewards, most participants selected *yes* for the high-p reward (81.25%) and *sometimes* for the low-p reward (50%).

## Discussion

We conducted this study for two primary purposes: (1) to determine whether a direct group SPA procedure would identify a preference hierarchy for groups of students with disabilities and (2) to evaluate whether the group SPA would accurately identify reinforcers for increasing desirable behavior in the context of a group contingency. A special education teacher implemented the procedures, and participants included two classrooms of middle school students with various disabilities (e.g., ASD, SLD). The group SPA used a PS format in which electronic pictorial options (i.e., free time activities) were presented on an interactive whiteboard and students selected their preferred option using online survey software. Students participated in the SPA after receiving brief directions and sessions required about 15 min each (45 min total for 3 sessions). We aimed to validate the group SPA results by alternating group contingency sessions in which high-p. versus low-p consequences were available contingent on students being prepared for class. We also collected data on student responses to a daily social studies quiz and administered a social validity survey.

### Primary Research Questions

Our first research question asked whether the group SPA would result in a hierarchy of participants’ preference for potential reinforcers, and a hierarchy emerged for both classes based on the data aggregated from three sessions. In Classroom One, the high-p choice received 17 more votes than the second choice (23.86% versus 21.10% of votes) and 120 more votes than the low-p choice (23.86% versus 4.38% of votes). There was a smaller margin between the two highest-preferred options in Classroom Two (21.31% versus 20.17% votes); however, the overall data pattern was similar to Classroom One with votes for the remaining options clearly distributed across a hierarchy.

These SPA results correspond with previous research on individually administered SPAs indicating that for some individuals, immediate access to the chosen stimulus may not be necessary for identifying preferences (Brodhead et al., 2019; Daly et al., 2009), and electronic pictures can replace tangibles (Brodhead et al., 2016). To our knowledge, this is one of few studies to evaluate a direct group SPA procedure and the first to include students with disabilities. Our procedures differed from previous group SPA research in that we used a PS format rather than a multiple stimulus format. We also did not provide immediate access to the chosen option (as in Layer et al., 2008 and Radley et al., 2019), which allowed us to feasibly include activity-based reinforcers. Finally, we omitted SPAs for individual students given that research by Layer et al. and Radley et al. indicated individual and group results would correspond, and our ultimate goal was to evaluate group SPA results in the context of a group contingency.

Our second research question asked whether student behavior would differ during group contingency sessions in which high-p consequences were available compared to sessions in which low-p consequences were available. Our data suggest a functional relation for both classes, given that the mean level of students prepared for class was elevated in high-p conditions compared to low-p conditions. However, both the high-p and low-p conditions increased desirable behavior compared to baseline. Additionally, our ATD designs were relatively brief and data in the low-p condition demonstrated possible increasing trends. These data patterns suggest the low-p condition may have become more effective over time, perhaps due to its alternation with the high-p condition (i.e., multiple-treatment interference) or due to students contacting the programmed contingencies across both conditions (i.e., completing an academic task instead of putting their heads down).

To our knowledge, this is the first study to attempt validation of group SPA results in the context of a behavioral intervention. Our results suggest that even a low-p consequence may increase desirable behavior during a group contingency, which aligns with systematic reviews that identified overall positive results of group contingencies even without the use of direct SPAs (Maggin et al., 2012; Pokorski et al., 2017). Nonetheless, our results also indicate that teachers may achieve the greatest increases in desirable behavior by identifying a highly preferred consequence to include in a group contingency.

### Secondary Questions

We also collected data on the percentage of correct responses to a daily video quiz in an effort to describe whether study conditions were associated with academic responding (i.e., whether being prepared for class resulted in student learning). Overall, we did not observe clear functional relations between the intervention conditions and quiz performance. It is important to note that the teacher wrote a unique quiz question for every session, which could account for the variability in these data across sessions. Although we aimed to have similar difficulty across questions, we cannot confirm whether that was the case. Student responses may have also been impacted by their level of background knowledge or interest in the daily news video. Future studies could further explore the impact of preparedness on academic responses by better controlling for these factors. Finally, we included a survey to determine whether the intervention procedures were motivating and enjoyable from students’ perspectives. These data indicated that students felt most motivated when the high-p choice was available and most enjoyed the high-p sessions compared to both baseline and low-p sessions.

### Limitations

Readers should note several limitations of this study. First, our group contingency package included several potentially effective components (e.g., BST for the target behavior, multiple daily reminders of expectations, assignment of an instructional activity to students who did not meet expectations), and we cannot be sure which of the components were necessary and sufficient to increase the percentage of students prepared for class. Second, our dependent variable was a brief, discrete behavior. We do not know how the group contingency would have impacted a more effortful, continuous behavior (e.g., engagement throughout the lesson). We chose this dependent variable because it was a common classroom concern and the teacher and instructional assistant could feasibly collect data during instruction; however, researchers may want to include continuous behaviors in future studies. Third, because we did not collect IOA data for Classroom Two during baseline, we cannot ensure the accuracy of data within that phase. Notably, IOA was 100% for nearly all sessions in which we did collect secondary data and we collected sufficient data during the experimental ATD phase for Class Two. Fourth, we did not measure the social validity of the group SPA procedures from students’ perspective (i.e., our survey only included questions related to the group contingency). Future research may investigate whether group SPA procedures like the ones in this study are enjoyable to students. Last, we were unable to complete an ABAB design for Classroom Two due to multiple interruptions (e.g., winter break) during the study timeline, and thus our evaluation of the group contingency in the second class is not as strong as our evaluation in the first class.

### Implications for Research

Several variables related to the development of group SPAs warrant future investigation. First, researchers should identify the number of group SPA sessions necessary to identify consistent results and whether the number of sessions should vary based on participant characteristics. Although standard PS procedures include only one session (Fisher et al., 1992), we included three sessions administered across three days, which we hypothesized would allow us to identify accurate and stable preferences for extended use during the group contingency. However, our session-by-session analyses suggest that all three sessions may not have been necessary. Notably, we generally identified the same high-p and low-p stimuli across all three sessions, suggesting that the group contingency would have used the same reinforcers if a single session had been conducted. This finding stands in contrast to similar analyses of individually administered MSWO assessments (Conine et al., 2021), which suggest that an aggregate measure of multiple SPA sessions may identify different highest-preferred stimuli than single SPA sessions. Such findings may be related to the differences between group and individual SPA formats, between PS and MSWO formats, or both. These finding should be replicated in future research.

Relatedly, we used results of the group SPA conducted at the beginning of the study during all sessions; future research could compare results of a pre-intervention SPA to a brief SPA included at the beginning of group contingency sessions (as Radley et al. (2019) also suggested). Additionally, researchers should identify whether other direct SPA formats can be effectively adapted for groups of students with disabilities. We chose a PS format because it limited the number of options available at once and allowed the teacher to create a presentation with all slides before the study (i.e., it was feasible). However, an electronic MSWO format is potentially more efficient if the implementor could create one slide with all options and then delete chosen options during the assessment (similar to Brodhead et al., 2016).

Researchers should also continue investigating methods for students to choose an option during group SPA sessions. We used Google Forms to create an anonymous voting method in alignment with previous group SPA research (Layer et al., 2008; Radley et al. 2019; Resetar Volz & Cook, 2009). We hypothesized that a public voting method (e.g., hand raising) would result in some students’ opinion influencing other students. However, we do not know whether this was necessary, and using the Google Form required time to analyze results outside of the sessions. Future research could investigate whether there are differential results during group SPA sessions that use public versus private responses.

This study also adds to the currently limited body of research on independent group contingencies, and our findings point to several avenues of future research on this intervention. For example, we suggest that future researchers consider whether some students need a unique reinforcer during independent group contingency sessions (Chow & Gilmour, 2015; Radley et al., 2019). In this study, 100% of students in Classroom One were prepared during the final high-p sessions, which suggests the same consequence was a reinforcer for all students’ behavior in that class. However, there was no group contingency session in Classroom Two during which 100% of students were prepared. Future researchers may choose to conduct an individual SPA with non-responders, evaluate correspondence of individual SPA results with the group SPA, and evaluate the effectiveness and social validity of including differentiated reinforcers within independent group contingency sessions. Additional research comparing the effectiveness of independent contingencies to interdependent and dependent contingencies is also warranted. Although some evidence suggests they can be equally effective (Groves & Austin, 2017), it is possible that an interdependent contingency would have yielded better outcomes in Classroom Two.

Finally, in future empirical evaluations, we encourage researchers to make procedural considerations to allow teachers to feasibly conduct group SPAs. In this study, a teacher was the primary implementer and data collector; thus, we prioritized her ability to implement procedures when designing them. For example, our use of the PS format aligned with her preference to create one set of materials and analyze results after administration, and the use of Chromebooks and Google Forms further increased the feasibility of data collection. From both an implementation and data collection standpoint, procedures that are feasible for teachers can increase the probability of accurate and sustained implementation in natural settings. Future research should also assess social validity from the implementer’s perspective.

## Recommendations for Practitioners

We believe a group SPA is a relatively simple procedure for special education teachers to implement. Nonetheless, special educators should note that assessment procedures used in this study are best suited for students who have low support needs (e.g., can follow spoken and written directions, can respond to an electronic survey). Special educators working with students with high support needs should likely continue using individual SPA procedures similar to those described by Fisher et al. (1992) or DeLeon and Iwata (1996).

If teachers do implement a group SPA, we recommend considering a few adaptations to our procedures. First, teachers who have limited time available may choose to implement one SPA session and then consider additional sessions only if they are unable to identify a highly preferred stimulus. Second, teachers who do not have access to or familiarly with the technology used in this study could use low-tech materials. For example, teachers could use printed pictures or tangible objects instead of slides, and even teachers who display options via technology may choose a low-tech response option for students (e.g., paper slips instead of electronic surveys). Combining results from three Google Forms required about 30 min and working knowledge of using formulas within spreadsheets; thus, teachers may prefer paper-based options. We do recommend that, until research indicates otherwise, teachers use an anonymous voting method to avoid students’ opinions being influenced by their peers. Last, we recommend teachers who use group SPAs to design a group contingency evaluate the effect of using a high-p reward on student behavior by comparing at least three sessions of baseline data to at least five sessions of intervention data (a simple A–B design). Similar to our results for Classroom Two, teachers may find that a small number of students do not meet expectations when a group reward is available, and those students may benefit from an individualized reward or a different type of contingency.

## Conclusion

This study adds to a small but growing body of literature indicating traditional SPA procedures can be efficiently and effectively scaled up for group contexts. Our results indicate that a PS group SPA may accurately identify reinforcers for increasing desirable behaviors of students with disabilities during group contingency sessions, and that the inclusion of group SPA results within a group contingency is socially valid from students’ perspectives. Although we identify several avenues for ongoing research on this topic, we encourage special educators who work with similar populations to replicate and adapt our procedures.

## Supplementary Information

Below is the link to the electronic supplementary material.

Supplementary file1 (DOCX 28 KB)

## Data Availability

The author confirms that dependent variable data generated and analyzed during this study are included as a supplemental file with this submission.
