# Supplementary material for: Using a Group Stimulus Preference Assessment to Design an Effective Group Contingency
Source: Behav Anal Pract. 2024 Oct 17;17(4):1008–22. doi: 10.1007/s40617-024-01003-2 (PMC11707153; doi:10.1007/s40617-024-01003-2)
Supplement: Supplementary file 1 — Supplementary file1 (DOCX 28 KB) [file 40617_2024_1003_MOESM1_ESM.docx]

Supplemental Table 1

*Classroom One Group Stimulus Preference Assessment Summary Data*

| Letter | Options | Total Votes | % Votes |
| --- | --- | --- | --- |
| B | Chromebook Freetime | 147 | 21.62 |
| A | Blooket | 130 | 19.12 |
| D | Heads-Up/7-Up | 108 | 15.88 |
| C | Drawing | 79 | 11.62 |
| F | Walk Track | 77 | 11.32 |
| NA | Neither | 64 | 9.41 |
| E | Sorting | 48 | 7.06 |
| G | Head Down | 27 | 3.97 |

*Note.* Data represent the number and percentage of votes each choice received across three sessions. NA = not applicable.

Supplemental Table 2

*Classroom Two Group Stimulus Preference Assessment Summary Data*

| Letter | Options | Total Votes | % Votes |
| --- | --- | --- | --- |
| A | Blooket | 75 | 19.74 |
| B | Chromebook Freetime | 71 | 18.68 |
| F | Walk Track | 58 | 15.26 |
| D | Heads-Up/7-Up | 54 | 14.21 |
| C | Drawing | 43 | 11.32 |
| E | Sorting | 36 | 9.47 |
| NA | Neither | 28 | 7.37 |
| G | Head Down | 15 | 3.95 |

*Note.* Data represent the number and percentage of votes each choice received across three sessions. NA = not applicable.

Supplemental Table 3

*Classroom One Group Contingency Data by Session*

| Session | # Students Not Prepared | # Students Prepared | BL % | High-P % | Low-P % | IOA % | Fidelity % |
| --- | --- | --- | --- | --- | --- | --- | --- |
| 1 | 10 | 0 | 0 | - | - | 100 | - |
| 2 | 10 | 0 | 0 | - | - | - | - |
| 3 | 11 | 0 | 0 | - | - | - | - |
| 4 | 10 | 0 | 0 | - | - | - | - |
| 5 | 10 | 0 | 0 | - | - | - | - |
| 6 | 2 | 9 | - | 81.81 | - | 100 | - |
| 7 | 1 | 9 | - | 90 | - | 100 | - |
| 8 | 3 | 8 | - | - | 27.27 | 100 | - |
| 9 | 0 | 11 | - | 100 | - | - | - |
| 10 | 2 | 8 | - | - | 80 | - | 100 |
| 11 | 0 | 12 | - | 100 | - | - | 100 |
| 12 | 1 | 9 | - | - | 90 | - | - |
| 13 | 10 | 1 | 9.09 | - | - | 100 | - |
| 14 | 10 | 1 | 9.09 | - | - | - | - |
| 15 | 11 | 0 | 0 | - | - | - | - |
| 16 | 2 | 9 | - | 81.82 | - | 81 | - |
| 17 | 2 | 9 | - | - | 81.82 | - | - |
| 18 | 2 | 8 | - | - | 80 | 100 | 100 |
| 19 | 0 | 11 | - | 100 | - | - | 100 |
| 20 | 1 | 10 | - | - | 90.90 | - | - |
| 21 | 0 | 11 | - | 100 | - | - | - |

Note. BL = baseline; High-P = group contingency sessions with highest-preferred consequence; IOA = interobserver agreement; Low-P = group contingency sessions with lowest-preferred consequence.

Supplemental Table 4

*Classroom Two Group Contingency Data by Session*

| Session | # Students Not Prepared | # Students Prepared | BL % | High-P % | Low-P % | IOA% | Fidelity% |
| --- | --- | --- | --- | --- | --- | --- | --- |
| 1 | 8 | 0 | 0 | - | - | - | - |
| 2 | 7 | 0 | 0 | - | - | - | - |
| 3 | 7 | 0 | 0 | - | - | - | - |
| 4 | 8 | 0 | 0 | - | - | - | - |
| 5 | 6 | 0 | 0 | - | - | - | - |
| 6 | 7 | 0 | 0 | - | - | - | - |
| 7 | 6 | 0 | - | - | 0 | 100 | 100 |
| 8 | 2 | 5 | - | 71.43 | - | 100 | 100 |
| 9 | 2 | 5 | - | 71.43 | - | - | - |
| 10 | 6 | 0 | - | - | 0 | - | - |
| 11 | 1 | 5 | - | 83.33 | - | - | - |
| 12 | 1 | 5 | - | - | 83.33 | - | - |
| 13 | 5 | 2 | - | - | 28.57 | - | - |
| 14 | 1 | 6 | - | 85.71 | - | - | - |

Note. BL = baseline; High-P = group contingency sessions with highest-preferred consequence; IOA = interobserver agreement; Low-P = group contingency sessions with lowest-preferred consequence.
